# Aneurism of the Brachial Artery from Venesection

**Published:** 1829-07-01

**Authors:** 


### ANEURISM OF THE BRACHIAL ARTERY FROM WOUND DURING BLEEDING.

Some interesting and instructive cases of the above accident, for accident we suppose it may be generally called, have been contributed by Mr. Smith, senior surgeon to the Bristol Hospital, in the first number of the Provincial Medical Gazette. The paper in question has oddly enough been headed, a "Letter on Blood-letting," though with ordinary blood-letting it has very little indeed to do.

*Case* 1. About two years ago, Mr. Smith was summoned to a patient in great haste, in consequence of an apprentice having opened the brachial artery with a lancet, in mistake for the vein. When Mr. S. arrived, the patient had lost a large quantity of blood, though the hæmorrhage had been arrested by the tourniquet. Mr. Smith cleaned the orifice and arm, and accurately closed the former by placing over it a small button-like compress, secured by adhesive straps and well regulated pressure. The tourniquet was then slackened, and the man sent to bed. The bowels were opened, and in two or three days there being some febrile excitement, the patient was copiously bled, but the arm was left undisturbed for a fortnight, when the pressure was removed. No tumour had appeared, an adhesive strap was re-applied pretty tight, to serve as a support, and from that time to this Mr. Smith has heard nothing of the patient.

*Case* 2. On the 15th of April, our author was summoned to a stout healthy workman, whose right brachial artery had been opened instead of the vein. The hæmorrhage had been very profuse, but a tourniquet was applied, and before Mr. Smith's arrival (three hours and a half after the accident) it was checked. The instrument had been screwed so tight that the man was in excessive torture, and the arm extremely swollen and livid. This case was treated in a similar manner with the former, and then the tourniquet gradually loosened when the circulation was restored, and the pain by degrees disappeared. The bandage was cautiously tightened from time to time, and on the 2d of May every thing was removed. No tumour existed, and the man was directed not to use the arm roughly, an adhesive strap being placed around it as a measure of precaution. Mr. Smith has since learned, that the man experiences no inconvenience in the part injured.

"I might relate other cases in my own practice or that of my friends, of the same kind, amply sufficient to shew that *well-regulated* pressure will generally succeed in preventing the formation of aneurism. Doubtless, occasionally it will fail, and we have had many cases of brachial aneurism sent to the infirmary from various quarters, in some of which very likely pressure had been properly applied."

The two cases above detailed were certainly more favourable cases for the employment of pressure than those where the artery is punctured through the vein. We can see no good reason why pressure, properly applied, should not prevent the egress of blood from, and effect the healing of a clean cut in a vessel so much under our command as the brachial. Reasoning, à priori, would naturally lead us to this conclusion, and the experience of surgeons, Mr. Smith's for instance, goes to establish its correctness. When, however, both vein and artery are wounded, pressure, however employed, will, beyond all doubt, be often unavailing in *preventing* the formation of one or other of the different species of aneurism which follow this particular injury, and if the aneurism be actually formed, it will very seldom suffice to *cure* it. Mr. Colles, we believe, of Dublin, succeeded in curing a circumscribed aneurism of the brachial artery after bleeding, but such cases are much too like angels' visits—few and far between. The following presented some unusual symptoms, and deserves to be recorded.

*Case* 3. A school-master, twentyfour years of age, of irritable temperament, residing the other side of the Severn, had his brachial artery wounded in bleeding, when the operator, aware of what had happened, bound a large quantity of lint on the orifice. The blood forced its way to the cellular membrane, and shortly afterwards, when the external wound was healed, a pulsating tumour made its appearance, and in the course of six weeks had acquired the size of a hen's egg. He was now admitted into the Bristol Infirmary, and next day the operation was performed of tying the brachial artery above. One of Mr. Smith's colleagues happening to have a finger on the tumour at the time that the incision was made through the integuments, informed him that the pulsation had instantly ceased on that being done. It was gone too from the wrist, although the pulsations in the other arm were strong, and the patient by no means in a state of syncope. The operation was continued and a ligature applied round what seemed to be the artery, but not secured, as no pulsation was presented by the vessel.

"For the purpose, of restoring the circulation, the arm was plunged into hot water, renewed from time to time for an hour; but our efforts were unsuccessful. The lips of the wound were now approximated a little with adhesive plaister, the ligature being left as before, and the man covered up warm, and placed on his bed by the fire of the consultation room, which opens into the operation theatre. All this occupied the space of time between one and half-past two. The man went to sleep, and was pretty comfortable, but the pulsation did not return till about seven o'clock, when it was pretty strong both in the tumour and at the wrist; and I had the satisfaction to find that a pull at the ligature checked it immediately and effectually. Assured thus that all was right, I made a knot, and tied the artery. The wound was then treated in the ordinary way, and my patient had a good night, and suffered but little.

"In nine days the ligature came away without a drop of blood, the wound was rapidly closing, and I flattered myself that all was well. Shortly after, however, he became attacked with erysipelas, spreading from the affected arm over nearly the whole of the trunk of the body, which occasioned suppuration in the cellular membrane of the limb, and sphacelus of some portion of its integuments; and considerably undermined his general health. When he was getting out of this, on the thirtieth day from the operation, there was a sudden and immense flow of blood through the wound where the vessel had been tied, which, in all probability, would have proved fatal, but for the accidental presence of a pupil. It was now judged expedient to amputate the arm, which I did immediately, a colleague commanding the artery by pressure, as there was no room for the tourniquet. The poor fellow bore it with more fortitude than I had anticipated, and after the operation said, 'Well sir, *now* I hope I shall get well.' In this hope you will readily believe I heartily joined, and I had indeed the pleasure of seeing him daily recovering for a fortnight or more, when he was suddenly, and without any ascertainable cause, seized with convulsions, and, in half an hour, a period was put to his sufferings.

"Upon examining the limb, we discovered that the brachial artery had ulcerated at the spot where the ligature had been applied. The aneurismal tumour was reduced to the size of a filbert."

This is certainly a puzzling as well as an unfortunate case, and the facts are all that are required, for commentary would be little else than conjecture. Mr. Smith, in concluding his "letter," mentions the particulars of the case of a young woman who plunged a sharp knife into the palm of her hand. A surgeon failed to discover the bleeding orifice, but by pressing with his single finger in various directions, Mr. S. at last hit upon one spot which commanded the hæmorrhage. Pressure on the bleeding surface itself, by means of a firm ball of rolled lint, compresses, and roller, was employed, and with perfect ultimate success.

Before leaving the subject of wounds of the palm of the hand, we may just allude to a case which occurred to Mr. Palmer, a very intelligent young surgeon, of Suffolk Place, and which has been published by him in the London Medical Gazette.

*Case.* W. Hall, æt. 28, a respectable tradesman, punctured with the point of a sharp leather knife, the ulno-carpal artery in the palm of the hand, about an inch beyond the pisiform bone. The wound was small but bled profusely; it was bandaged up for the ten following days, by which time it had perfectly cicatrized, and he took no further notice of it, until a few days afterwards he observed a small pulsating tumour in the site of the cicatrix. On the 9th of June, when Mr. Palmer saw him, the tumour was as large as a small walnut, and had a strong pulsation, which appeared to be daily increasing, and was not stopped except by the simultaneous compression of the ulnar and radial arteries. On the 12th of June, after having placed a tourniquet upon the arm, Mr. P. made an incision, about two inches and a half in length over the centre of the tumour, and immediately secured the ulnar artery where it entered into it. The sac was then opened, but on slackening the tourniquet a strong jet of blood issued forth, and the point of a small probe not readily discovering the opposite orifice of the vessel, the latter was dissected for externally, found, and secured. Still, however, notwithstanding the branches of supply were thus tied above and below, a considerable hæmorrhage issued from the sac, and on loosening the tourniquet a second time, and finding it impossible to discover the bleeding vessel in the bottom of the wound, Mr. P. passed a double ligature beneath the sac, and tied its two ends above and below, including the sac between the knots. The hæmorrhage was by this means completely arrested—the wound was lightly dressed—the last ligature taken away on the 30th, and on the 7th of the following month the cicatrix was perfect.

We fully agree with Mr. Palmer in thinking that the proceeding he adopted was infinitely preferable to tying the radial and ulnar arteries, because it struck at once at the root of the affection, was much more certain in its effects, and was one operation instead of two. It is quite a mistake to suppose that the ordinary operation for aneurism in the course of a large vessel is applicable to all the affections in the smaller ramifications, and so surgeons are beginning to find it.

---

